# Data-Driven Multi-Objective Optimization of Drilling Performance in Multi-Walled Carbon Nanotube-Reinforced Carbon Fiber-Reinforced Polymer Nanocomposites

**DOI:** 10.3390/polym18080986

**Published:** 2026-04-18

**Authors:** Hediye Kirli Akin

**Affiliations:** Department of Industrial Engineering, Osmaniye Korkut Ata University, 80000 Osmaniye, Turkey; hediyeakin@osmaniye.edu.tr

**Keywords:** CFRP nanocomposites, MWCNT, delamination factor, multi-objective optimization, NSGA-II, MOPSO

## Abstract

Carbon fiber reinforced polymer (CFRP) composites are widely used in many engineering applications such as aerospace, automotive, and defense industries due to their superior properties such as high specific strength, stiffness, and corrosion resistance. However, these materials require drilling, especially during assembly processes. Damage mechanisms arising during this process, such as delamination, high thrust force, and torque, negatively affect structural integrity and production quality. This study proposes a data-driven, multi-objective optimization approach to solve problems encountered during drilling in multi-walled carbon nanotube (MWCNT)-reinforced CFRP nanocomposites. The study considers the MWCNT reinforcement ratio, cutting speed, and feed rate as process parameters and examines their effects on thrust force, torque, and delamination factor. Second-degree polynomial regression-based prediction models were created using the experimental data obtained, and these models were included in the multi-objective optimization process. During the optimization phase, thrust force and torque values were simultaneously minimized, while the delamination factor was kept below the statistically determined constraint of *F_d_* ≤ 1.054. Pareto-optimal solution sets were obtained using NSGA-II and MOPSO meta-heuristic algorithms in the solution process. The results indicate that suitable combinations of drilling parameters can be identified through Pareto-based optimization, allowing significant reductions in thrust force and torque while maintaining the delamination factor below the specified limit. The study presents a reliable optimization approach for the more efficient machining of CFRP nanocomposites.

## 1. Introduction

Carbon fiber-reinforced polymer (CFRP) composites have redefined engineering standards in the aerospace, automotive, and defense industries with their superior qualities such as high specific strength, rigidity, and corrosion resistance [1]. The long-term reliability and structural integrity of advanced engineering systems heavily depend on understanding and mitigating internal damage mechanisms. Recent studies emphasize the critical importance of predicting defect propagation, micro-structural degradation, and the probabilistic nature of damage in heterogeneous structural systems subjected to complex loading conditions [2]. To enhance structural resilience and load-bearing capacity under severe stress states, the integration of fiber-reinforced polymer (FRP) composites and advanced hybrid reinforcement strategies has been widely adopted as a highly effective design approach [3]. However, the practical manufacturing and assembly of these high-performance FRP structures inevitably require secondary machining processes, such as precision drilling. If the cutting parameters are not accurately modeled and optimized, drilling operations can introduce severe mechanical defects, which drastically reduce the anticipated structural reliability of the composite components. The heterogeneous and anisotropic nature of CFRP triggers serious damage mechanisms during drilling, such as fiber breakage, rapid tool wear, rough inner surfaces, and delamination, defined as interlayer separation, endangering structural integrity and fatigue life [4].

In recent years, incorporating nanoscale fillers into CFRPs has emerged as a primary strategy to meet the performance demands of modern engineering applications. Specifically, literature from 2025 indicates a strong trend toward using multi-walled carbon nanotubes (MWCNTs) to develop advanced structural parts, particularly for the aerospace sector [5]. This nanoscale addition goes beyond simple mechanical reinforcement; it introduces multifunctional properties to the laminates [6] and fundamentally alters the damage mechanisms and erosion resistance of these hybrid structures under severe conditions [7]. Given these material advancements, achieving high-quality holes during secondary machining processes like drilling is essential to maintain the structural integrity of these new-generation nanocomposites.

In the literature, delamination is generally associated with the “push-out” mechanism, which occurs when the axial thrust force generated during drilling exceeds the interlaminar bond strength. Pioneering work in this field by Hocheng and Dharan (1990) analytically modeled the mechanical origins of delamination and demonstrated the decisive role of axial force in this damage [8]. Subsequently, the effects of cutting speed and feed rate on these forces were studied in detail; it was observed that low feed rates suppressed delamination but were not sustainable in terms of manufacturing efficiency. At this point, researchers turned to nano-reinforcements such as MWCNT to strengthen the matrix structure. The “nanobridging” effect established by MWCNTs within the matrix is thought to significantly reduce both thrust forces and damage formation by preventing crack propagation. However, the MWCNT reinforcement ratio, matrix–fiber–nanofiller interface interactions, and complex interactions between processing conditions make drilling behavior a highly variable and nonlinear problem that is difficult to solve [9].

As stated by Abrao et al. in 2007 and Davim and Reis in 2003, most of the existing studies on the optimization of CFRP drilling processes focus on only a single performance criterion [10,11]. These approaches, which generally concentrate on the delamination factor or axial force, often overlook the complex trade-offs inherent in the drilling process. However, it is known that parameter combinations that provide low delamination can lead to high cutting forces, which is disadvantageous in terms of tool life and energy efficiency [12]. In this context, CFRP drilling operations should be considered a nonlinear and multidimensional problem requiring the simultaneous optimization of conflicting objectives.

Taguchi designs, full factorial experiments, analysis of variance, response surface methodology (RSM), and multi-criteria decision making techniques are widely used for optimizing the drilling parameters of CFRP nanocomposites [13]. In recent years, the integration of nanoscale reinforcements into matrix structures has emerged as a highly effective strategy to mitigate drilling-induced damage. For instance, recent studies have demonstrated that the addition of carbon nanotubes (CNTs) significantly reduces delamination and thermal loading during the drilling of composite laminates by enhancing the interfacial shear strength [14]. Furthermore, the pursuit of optimal hole quality and the minimization of machining defects through systematic statistical and optimization techniques remain a primary focus across various advanced structural materials, including metal matrix composites [15]. Although meta-heuristic methods offer powerful tools for solving engineering problems, studies on the integration of these methods with material-oriented physical damage mechanisms are quite limited in the literature. This study aims to fill this gap by investigating the drilling performance of MWCNT-reinforced CFRP nanocomposites using data-driven models and advanced optimization techniques. Unlike previous research, the delamination factor (*F_d_*) is defined as an engineering constraint (*F_d_* ≤ 1.054) based on the first quartile (*Q*_1_) value of the experimental data distribution, rather than a directly minimized objective function. Second-order regression models created from data obtained from experiments conducted with uncoated carbide drills were integrated into NSGA-II and MOPSO algorithms to identify Pareto-optimal solution sets. In this regard, the study aims to provide a scientific guide for the damage-free and efficient processing of complex composite structures by interpreting the algorithmic results from a material behavior perspective.

## 2. Materials and Methods

### 2.1. Production and Experimental Design of CFRP Nanocomposites

**Figure 1:**
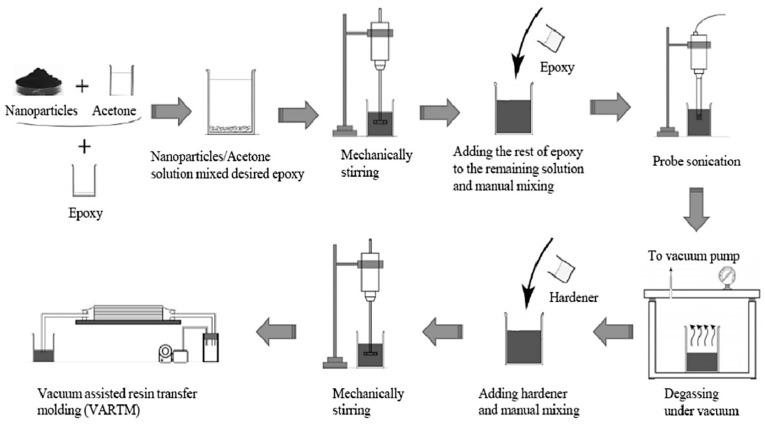
Flowchart of the production process for MWCNT-reinforced CFRP composite plates [9]. Arrows represent the direction of the production workflow.

The nanocomposite samples used in this study were obtained through a service contract with Innovative Material Technologies Industry and Trade Ltd. (Izmir, Turkey). The production process of the samples was carried out in accordance with the company’s standardized, high-precision protocols, as shown in Figure 1:

In accordance with our experimental design, the MWCNT to be added to the epoxy matrix were determined at concentrations of 0.5% and 1.0% by weight. Additionally, the production of reference samples containing no nanoparticles (0% MWCNT) was also provided by the same company under the same conditions.

In the MWCNT-reinforced groups, a multi-step dispersion procedure was applied by the company to prevent the agglomeration of the nanotubes within the resin and to achieve a homogeneous distribution. In this context, the MWCNTs were first mechanically mixed in acetone, then added to the epoxy-hardener mixture and processed for 60 min using a probe-type ultrasonicator. To completely remove the acetone from the structure, the mixture was left in an 80 °C vacuum oven for 24 h, after which the plates were moved to the molding stage.

Carbon fiber-reinforced polymer (CFRP) plates were manufactured in-house using the Vacuum-Assisted Resin Transfer Molding (VARTM) method. The plates were constructed from 8-ply unidirectional carbon fiber fabrics with a fiber volume fraction of approximately 55%. They were then cured at room temperature under 1.6 MPa pressure for 24 h. The composite sheets were cut into samples measuring 80 × 100 mm and 7 ± 0.2 mm thick for use in drilling tests.

The drilling tests were conducted on a CNC vertical machining center. The thrust force (*F*) and torque (*T*) values generated during the process were recorded in real time using a Kistler 9257B dynamometer connected to a Kistler Type 5070 charge amplifier, and the obtained data were processed using DynoWare (version 2.4.1.3) software [9].

**Figure 2:**
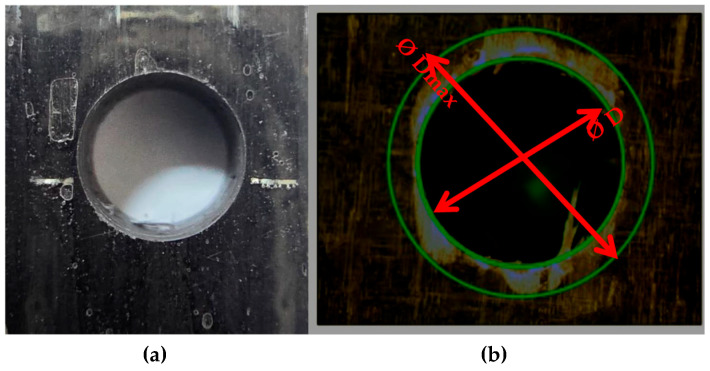
Evaluation of delamination damage using optical microscopy: (**a**) A representative magnified view of the damaged area and the edges of the hole; (**b**) Steps for determining the nominal drill diameter (*D*) and the maximum damage diameter (*D_max_*).

Measurements of the delamination damage resulting from the drilling process were performed using a Euromex Holland Type PB 4161 optical microscope. To increase measurement accuracy and prevent errors caused by optical misalignment, each drilled hole was photographed from different angles. The maximum delamination diameter (Dmax) and nominal hole diameter (D) were measured three times for each sample, and the standard deviations of these measurements were calculated to determine the margin of error. Figure 2a illustrates a magnified view of the characteristic delamination zone formed during the drilling process, while Figure 2b depicts the optical assessment methodology and the identification of the diameters used to calculate the delamination factor.

### 2.2. Force, Torque, and Delamination Measurement

The mechanical responses generated during the drilling process are the most fundamental factors determining the machinability characteristics of CFRP composites. The thrust force measured in this context represents the fundamental axial load component generated along the drill axis during drilling, which plays a decisive role in the formation of delamination in layered CFRP laminates. Torque, on the other hand, reflects the torsional resistance arising during the interaction between the drill and the material, providing critical information about the energy requirements and tool stress of the process [16].

The piezoelectric signals obtained from the dynamometer (Kistler 9257B) were transferred to the computer environment in real time via DynoWare software and analyzed digitally. The thrust force and torque values obtained were used as key performance indicators to evaluate the machinability behavior of composites during the drilling process and to feed the multi-objective optimization model in the subsequent stages of the study.

One of the most critical damage mechanisms encountered during the drilling of layered composite materials is delamination. This damage occurs at the hole entry and exit regions, negatively affecting the machining quality and structural integrity of the part. In this study, the delamination factor (*F_d_*), a widely accepted criterion in the literature, was used to quantitatively evaluate the amount of delamination [17].

To ensure high precision in damage assessment, the delamination at the hole entry and exit was characterized using a high-resolution optical microscope. To minimize measurement errors and ensure statistical reliability, each specimen was evaluated three times at different orientations, and the average values were recorded. The identification of the nominal drill diameter (*D*) and the maximum damaged diameter (*D_max_*) is schematically illustrated in Figure 2. As shown in Equation (1), the delamination factor (*F_d_*) is defined as the ratio of the maximum diameter of the damaged area to the nominal drill diameter [18]:

(1) $$F_{d}=\frac{D_{max}}{D}$$

Following the drilling process, detailed examinations conducted on the samples evaluated the damaged areas formed in both the entry and exit regions. Higher delamination factor values indicate more severe surface damage and interlayer separation. This parameter is also considered a reliable criterion by researchers such as Chen (1997) and Abrão et al. (2007) in defining drilling-induced damage in layered composite materials [10,18]. The obtained delamination data were used as a fundamental constraint parameter both in training the surrogate models and in controlling the delamination occurring at the output stage during the multi-objective optimization process.

### 2.3. Experimental Parameters and Measurements

In this study, a multi-level experimental design was applied to comprehensively investigate the parameters affecting the drilling process of CFRP nanocomposites using only uncoated carbide drills (Ø8 mm). The control factors in the experimental design were selected as the MWCNT weight ratio in the composite, cutting speed, and feed rate. Each parameter was evaluated at three different levels to investigate their effects on the thrust force, torque, and delamination behavior during the drilling process. The experimental parameters and their specific levels are presented in Table 1.

The experimental combinations created were applied in a random order to prevent potential systematic errors. A full factorial experimental design approach was adopted to evaluate not only the individual effects of the factors but also the binary and multiple interactions between them. All data obtained from the experiments were pre-evaluated to ensure consistency and were organized as the primary input for subsequent data-based modeling and multi-objective optimization studies.

Following the drilling operations, examinations were conducted to evaluate the damage at both the hole entry and exit regions. The delamination behavior was quantitatively expressed using the delamination factor. To ensure high measurement precision and minimize experimental error, three separate measurements were taken for each sample using a high-resolution optical microscope, and the average values were used for final calculations.

### 2.4. Data-Driven Constrained Multi-Objective Optimization Framework

The drilling of CFRP nanocomposites is inherently a nonlinear multi-objective problem due to the complex interactions between the material’s anisotropic structure and cutting parameters [19]. In this study, an optimization model was designed to increase process efficiency (minimization of thrust force and torque) while preserving structural integrity (delamination constraint).

#### 2.4.1. Surrogate Model Development

To apply multi-objective optimization algorithms, mathematical models were first developed based on experimental data. These regression-based predictive models estimate the delamination factor, thrust force, and torque as outputs, using reinforcement ratio, cutting speed, and feed rate as input variables. The developed models were directly used in the optimization analyses. To represent the complex relationships between process parameters and machining responses, second-order multivariate polynomial regression models were constructed following the approach proposed by Montgomery [20]. For the torque model, a stepwise regression method based on statistical significance (*p* < 0.05) was applied to improve model robustness. In contrast, the thrust force and delamination models were retained in their full second-order form to preserve interpretability. Insignificant interaction and quadratic terms were removed where necessary to prevent overfitting and improve the adjusted R^2^ values.

(2) $$y=\beta_{0}+\beta_{1}w+\beta_{2}v+\beta_{3}f+\beta_{11}w^{2}+\beta_{22}v^{2}+\beta_{33}f^{2}+\beta_{12}wv+\beta_{13}wf+\beta_{23}vf+\varepsilon$$

In Equation (2), y represents the response variable (delamination factor, thrust force, or torque), *w* represents the weight ratio, *v* represents the cutting speed, *f* represents the feed rate, *β_i_* represents the regression coefficients, and *ε* represents the error term. The performance of the developed prediction models was evaluated using the determination coefficient (R^2^), adjusted determination coefficient ($R_{adj}^{2}$), and root mean square error (RMSE) criteria. High determination coefficients indicate that the developed surrogate models have achieved a sufficient level of accuracy for optimization.

As summarized in Table 2, the R^2^ values for all drilling responses range from 0.64 to 0.93, indicating that the models can explain a significant portion of the total variance in the experimental data. In particular, the thrust force model exhibited the highest prediction accuracy with a high R^2^ value of 0.93 and a low RMSE value of 2.56 N.

The significance values (*p*-value) calculated for all regression models were well below the conventional threshold of 0.05 (*p* < 0.01). This confirms that the relationship between the independent variables (*w*, *v*, *f*) and the drilling outputs is statistically significant and that the results are not random. The RMSE value of 0.03 obtained for the delamination factors ensures that the models used capture the complex fracture mechanisms of MWCNT-reinforced CFRP composites with high accuracy. These statistical results demonstrate that they are suitable and robust for use as an objective function in the next stages, which are the multi-objective optimization processes. Accordingly, the multi-objective optimization model of the study is defined below:

(3) $$w\in \;\left[0,\;1.0\right],\;v\in \;\left[25,\;75\right],f\in \;\left[0.1,\;0.2\right]$$

(4) $$\min Z(w,\;v,\;f)=\left\{F\left(w,v,f\right),\;T\left(v,f\right)\right\}$$

(5) $$F_{d}\left(w,\;v,\;f\right)\leq 1.054\;0\leq w\leq 1.0\;\;\left(\%\right)\;25\leq v\leq 75\;\;\;\;m/min\;0.1\leq f\leq 0.2\;mm/rev$$

The decision variables of the multi-objective optimization problem are given in Equation (3), the objective function in Equation (4), and the constraints in Equation (5). Unlike previous studies, delamination has been redefined within a framework where it is treated as an engineering constraint rather than a direct objective function. The delamination factor *F_d_* is not directly minimized as an objective but is treated as an engineering safety constraint. This value is based on the first quartile (*Q*_1_) statistic of the experimental distribution. The delamination limit value was determined using a statistical threshold based on the experimental data distribution. All *F_d_* values from the experiments were sorted from smallest to largest, and the first quartile (*Q*_1_) value was calculated. Where *n* is the total number of experiments, *Q*_1_ represents the lower 25% of the data distribution and is calculated according to Equation (6).

(6) $$Q_{1}=F_{d\;\left[0.25(n+1)\right]}$$

**Figure 3:**
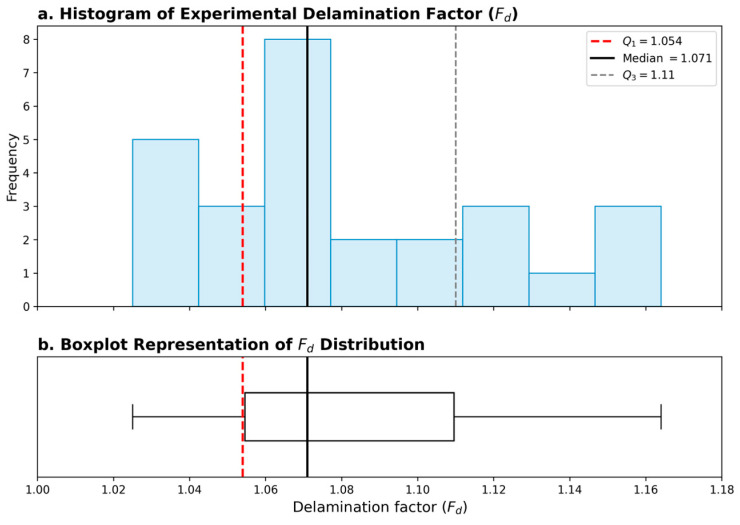
Statistical distribution of experimental delamination factor (*F_d_*) values. (**a**) Histogram with quartile boundaries (*Q*_1_, *Q*_2_, *Q*_3_); (**b**) Boxplot representation of the same dataset. The first quartile (*Q*_1_ = 1.054) defines the upper limit of the lowest 25% damage region and is adopted as the engineering constraint in the optimization framework.

The *Q*_1_ value calculated according to Equation (6) was found to be 1.054, which represents the upper limit of the lowest damage zone obtained experimentally. This approach both guarantees damage control and allows for the investigation of feasible parameter combinations. The distribution of experimental *F_d_* values and their quartile limits are presented in Figure 3.

As shown in Figure 3, the *Q*_1_ value naturally forms a threshold that separates the low damage region within the distribution. When the histogram and box plot are evaluated together, it is clear that *Q*_1_ represents the lower quartile of the data set and determines the upper limit of the highest quality production region obtained experimentally.

The second-degree multivariate polynomial regression equations based on experimental data, which form the objective functions (*F* and *T*) and constraint function (*F_d_*) of the multi-objective optimization algorithm, are detailed below:

(7) $$\begin{array}{c} F(w,v,f)=26.416-14.867w-0.058v+447.5f+2.876w^{2}+0.001v^{2}\\ \;\;\;\;\;\;\;\;\;\;\;\;\;\;\;\;\;\;\;\;\;\;\;\;\;\;\;\;-791.778f^{2}+0.065wv+9.6wf-1.368vf \end{array}$$

(8) $$T(v,\;f)=9.68+252.062f+0.003v^{2}-2.918vf$$

(9) $$\begin{array}{c} F_{d}(w,v,\;f)=1.257-0.189w-0.001v-1.701f+0.075w^{2}+5.733f^{2}\\ \;\;\;\;\;\;\;\;\;\;\;\;\;\;\;\;\;\;\;\;\;\;\;\;\;\;\;\;\;\;+0.001wv-0.16wf+0.006vf\;(Constraint) \end{array}$$

The mathematical models obtained for thrust force (*F*) and torque (*T*) are expressed by Equations (7) and (8), respectively; while the constraint function developed for the delamination factor (*F_d_*), defined as a limit value in the optimization process, is detailed in Equation (9). The coefficients of the developed regression models and the statistical performance indicators reflecting the predictive power of the models are presented comparatively in Table 3.

As shown in Table 3, the high R^2^ values, particularly in the *F* and *T* models, ensure a strong correlation between experimental data and mathematical estimates, as well as the reliability of the models for the optimization process. When examining the coefficients, the positive and high coefficients of the feed rate (*f*) indicate that it is the most dominant factor in increasing both force and torque, while the negative coefficients of the MWCNT weight ratio (*w*) indicate that nanotube reinforcement reduces thrust force and delamination damage. These statistical findings demonstrate that the model provides a solid foundation for multi-objective optimization.

#### 2.4.2. Multi-Objective Optimization Strategy

To scan the solution space of the multi-objective optimization model detailed and constrained in Section 2.4.1, two different meta-heuristic algorithms were used: The reason for using two different approaches is to confirm the accuracy and algorithmic robustness of the obtained Pareto-optimal solutions. The first of these approaches is NSGA-II (Non-dominated Sorting Genetic Algorithm II), which was preferred because it provides a broad Pareto distribution in complex solution spaces through its “elitism strategy” and “non-dominated sorting mechanism” [21]. Secondly, MOPSO (Multi-Objective Particle Swarm Optimization) was selected based on its high convergence speed and its success in finding the best solutions thanks to its “external repository” structure [22].

The algorithm used in the NSGA-II approach basically follows these steps [21]:

i. The population is divided into layers according to the Pareto dominance relationship (non-dominated sorting).

ii. The crowding distance is calculated to preserve diversity for solutions within the same layer:

(10) $$CD_{i}=\sum_{k=1}^{m}\frac{f_{k}^{i+1}-f_{k}^{i-1}}{f_{k}^{max}-f_{k}^{min}}$$

As formulated in Equation (10), the crowding distance (*CD_i_*) serves as a crucial diversity-preserving mechanism. It estimates the density of solutions surrounding a particular individual by calculating the average distance to its two closest neighbors along each objective function. By incorporating this metric into the selection process, NSGA-II ensures a uniform distribution of the Pareto-optimal front, preventing the algorithm from converging onto a narrow region of the search space. This approach is particularly vital for the multi-objective optimization of drilling parameters, as it provides a broad spectrum of feasible trade-offs between thrust force and torque.

iii. The selection process is performed using a binary tournament based on dominance degree and accumulation distance.

iv. A new generation is created by applying crossover and mutation operators.

Applicable solutions for constrained problems are evaluated first, while inapplicable solutions are ranked according to the magnitude of constraint violation.

In the MOPSO approach, each particle evolves using the following velocity and position update equations [23]:

(11) $$v_{i}^{t+1}=\omega v_{i}^{t}+c_{1}r_{1}(p_{i}-x_{i}^{t})+c_{2}r_{2}(g-x_{i}^{t})$$

(12) $$x_{i}^{t+1}=x_{i}^{t}+v_{i}^{t+1}$$

As detailed in Equations (11) and (12), the movement of each particle in the search space is governed by its velocity and position updates. In these expressions, the search trajectory is influenced by the particle’s personal best position (*p_i_*), a global leader solution (*g*) selected from the external Pareto repository, and the inertia weight (ω) which balances exploration and exploitation. The stochastic nature of the search is facilitated by learning coefficients (*c*_1_, *c*_2_) and random numbers (*r*_1_, *r*_2_) uniformly distributed in U(0,1). This iterative update mechanism enables the swarm to effectively navigate the complex parameter space of CFRP machining to identify optimal drilling conditions.

At this stage, the basic strategy is to mutually verify the accuracy and stability of the Pareto sets obtained by utilizing the different search mechanisms of both algorithms. The control parameters used in running the algorithms are presented in Table 4.

The optimization process was executed via a custom Python script (Python 3.11.14, Anaconda Inc., Austin, TX, USA) based on the regression equations presented in Section 2.4.1. By comparing the solutions obtained from both algorithms, Pareto-optimal points were identified that simultaneously minimize thrust force and torque while satisfying the engineering constraint of *F_d_* ≤ 1.054. This dual-algorithm approach minimizes the risk of the individual methods becoming trapped in local optima and enhances the reliability of the findings.

## 3. Results

This section presents the findings obtained from the experimental study and the subsequent multi-objective optimization analyses. To provide a clear basis for the modeling and optimization stages, the complete set of experimental results is first presented. Table 5 summarizes the measured delamination factor (*F_d_*), thrust force (*F*), and torque (*T*) values corresponding to all combinations of drilling parameters.

The dataset in Table 5 forms the foundation for the development of the regression models and the constrained optimization framework. A preliminary examination of the results reveals that both feed rate and MWCNT reinforcement ratio have a pronounced influence on drilling performance, particularly on thrust force and delamination behavior. These observations are further explored through the optimization analyses presented in the following sections.

To clarify the effect of MWCNT reinforcement on drilling performance, the experimental results were comparatively evaluated for the three reinforcement levels (0%, 0.5%, and 1.0%), as summarized in Table 6. The results show that increasing the MWCNT content generally reduces thrust force and delamination, while torque exhibits a more moderate variation. The 0.5% level shows intermediate behavior, whereas the 1.0% condition provides the most favorable overall performance.

### 3.1. Multi-Objective Optimization and Pareto Front Analysis

**Figure 4:**
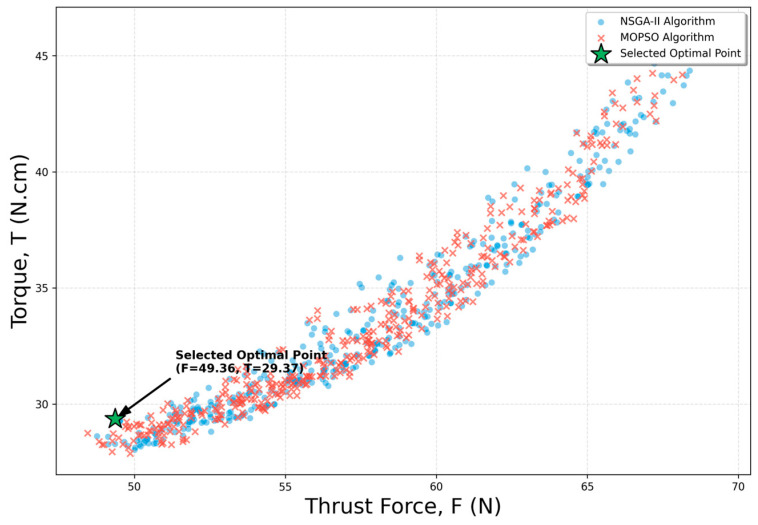
Pareto frontier analysis showing the trade-off balance between thrust force (*F*) and torque (*T*) (under the constraint *F_d_* ≤ 1.054). Blue points represent the outputs of the NSGA-II algorithm, while red markers represent those of the MOPSO algorithm.

The optimization process aimed to simultaneously minimize the thrust force (*F*) and torque (*T*) values, which are key performance indicators in the drilling operation of MWCNT-reinforced composites, while maintaining the delamination factor (*F_d_* ≤ 1.054) under a statistical constraint. The Pareto frontier graph presented in Figure 4 shows that both algorithms converged to a similar solution set and successfully captured the trade-off balance between the two objectives.

The best solution sets identified by the NSGA-II and MOPSO algorithms that fully satisfy the constraints are presented comparatively under different priority scenarios in Table 7.

As shown in Table 7, in the minimum thrust force (*F*)-focused scenario, the maximum MWCNT reinforcement ratio of 1.0% and the minimum feed rate (*f* = 0.1 mm/rev) resulted in a value of 48.03 N. This situation ensures that the maximum reinforcement ratio minimizes the vertical loads during drilling by increasing the strength of the matrix structure. On the other hand, in the minimum torque (*T*)-focused scenario analysis, it is observed that the optimum MWCNT ratio is balanced at around 0.84%, resulting in a value of 27.79 N·cm. This small difference is a result of the friction effect on torque caused by the increase in MWCNT content. In the high productivity (optimal) scenario, both objectives were attempted to be optimized simultaneously, and the solution was determined to be 0.93% MWCNT, a cutting speed of 70 m/min, and a feed rate of 0.1 mm/rev. At all these optimal points, the structural integrity of the material was maintained, and the delamination factor (*F_d_*) remained below the target limit of 1.054.

Figure 4 shows that the NSGA-II and MOPSO algorithms produce a very close and consistent Pareto frontier in the solution space. The tendency for torque values to increase in the region where thrust force is minimized reflects the typical trade-off nature of the drilling process.

In particular, the concentration in the lower-left region of the graph represents the optimal solution (*F* = 49.36 N, *T* = 29.37 N·cm), obtained at 0.93% MWCNT reinforcement, a cutting speed of 70 m/min, and a feed rate of 0.1 mm/rev. The overlap of both algorithms in this region indicates that the obtained optimal values are not merely mathematical estimates but are also physically consistent.

### 3.2. Performance Evaluation of the Algorithms

To quantitatively determine the effectiveness of the meta-heuristic algorithms used (NSGA-II and MOPSO) for this drilling problem, the Hypervolume (HV) and Spacing (S) metrics were employed.

i. Hypervolume (HV) Analysis: The HV metric, which measures the volume covered by the Pareto frontier, reflects both the convergence and diversity performance of the algorithms [24]. According to the results presented in Table 8, MOPSO achieved a slightly higher HV value (0.876) compared to NSGA-II (0.842), indicating a marginally better coverage of the solution space.

ii. Spacing (S) Analysis: The Spacing metric, which evaluates the uniform distribution of solutions on the Pareto frontier, was calculated as 0.114 for NSGA-II and 0.138 for MOPSO. Since lower values indicate better distribution, NSGA-II provided a more uniform spread of solutions along the Pareto front [25].

iii. Computational Time: In terms of algorithm efficiency, MOPSO completed the optimization process in 32.14 s, demonstrating faster performance compared to NSGA-II (42.85 s).

In conclusion, while MOPSO achieved a slightly higher hypervolume value, NSGA-II produced a more uniformly distributed Pareto front. On the other hand, MOPSO demonstrated superior computational efficiency. The consistency between the results of the two algorithms confirms the reliability of the proposed optimization framework. Comparative results for the performance metrics are presented in Table 8.

**Figure 5:**
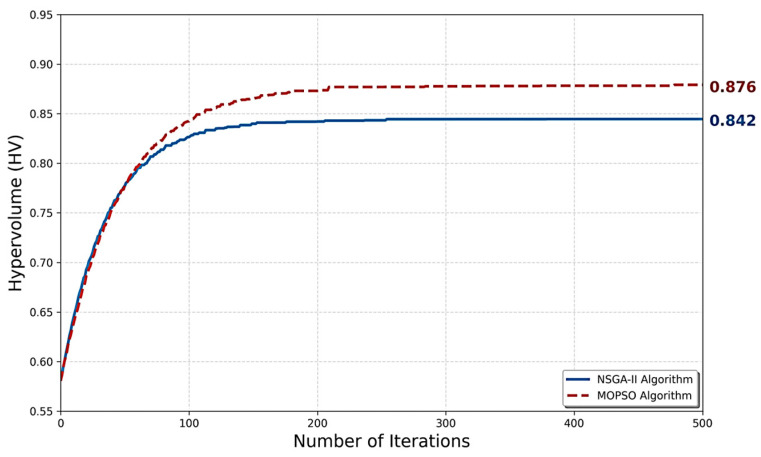
Comparison of the convergence performance of the NSGA-II and MOPSO algorithms over 500 iterations based on Hypervolume (HV) metrics.

The performance comparison is summarized in Table 8, while Figure 5 illustrates the convergence behavior of both algorithms.

Figure 5 shows that the NSGA-II and MOPSO algorithms exhibit a convergence curve that rises rapidly in the initial phase and then levels off. The MOPSO algorithm achieved a slightly higher hypervolume (HV = 0.876) compared to NSGA-II (HV = 0.842), indicating a marginally better coverage of the solution space.

The straight lines indicate that both algorithms reached a stable structure after approximately 300 iterations and successfully converged to the Pareto frontier.

### 3.3. Physical and Mechanical Analysis of Optimal Parameters

In this section, the individual and interactive effects of the MWCNT reinforcement ratio and feed rate on drilling performance are analyzed in light of regression models and optimization outputs.

Effect of MWCNT Reinforcement: It is observed that higher MWCNT reinforcement ratios (around 0.9–1.0%) tend to promote a “bridging” effect within the matrix structure, enhancing interlayer adhesion. This effect likely restricts the deformation occurring at the tool entry, thereby contributing to the reduction in thrust force and delamination.

Effect of Feed Rate (*f*): Regression equations confirm a positive correlation between feed rate (*f*) and thrust force (*F*). Lower feed rate values tend to reduce the axial load applied by the drill, thereby decreasing the risk of delamination. In contrast, higher feed rates can lead to exceeding the critical thrust force associated with push-out type delamination at the drill exit. The optimization results suggest that lower feed rate levels provide more favorable conditions for maintaining the delamination factor within the defined constraint.

### 3.4. Experimental Validation of Regression Models and Error Analysis

Validation experiments were conducted to verify the reliability of the developed regression models and to support the multi-objective optimization process. Within this scope, 7 different control experiments based on central composite design principles were performed. The experimental parameters were tested in a laboratory environment, and the actual measurements obtained were compared with the model predictions. A comparative validation matrix, including the prediction accuracy and error margins of the models on thrust force (*F*), torque (*T*), and delamination factor (*F_d_*), is presented in Table 9.

When examining the data in Table 9, it can be seen that the developed surrogate models provide strong prediction performance for all outputs. These results indicate that the models are reliable and can effectively support the optimization process. The error margins observed for thrust force (*F*) and delamination factor (*F_d_*) are generally low, confirming the predictive capability of the models. In particular, the delamination factor errors remain within acceptable limits across all experiments (e.g., 5.52% in Exp. 7), indicating that the model successfully captures the overall damage behavior of the composite material. For torque (*T*), relatively higher error values are observed in some cases (e.g., 20.48% in Exp. 3). However, this can be attributed to the anisotropic nature of the composite structure and the variability in chip formation during the drilling process, which are difficult to fully capture using polynomial regression models.

Furthermore, the consistency between the experimental and predicted trends also supports the reliability of the Pareto-optimal solutions obtained from the optimization process, indicating that the proposed approach can effectively guide practical parameter selection.

Due to experimental limitations related to composite fabrication, direct verification at the exact Pareto-optimal points was not possible. However, verification experiments conducted in near-optimal regions demonstrated a consistent agreement with model predictions, indirectly supporting the reliability of the optimal solutions.

## 4. Conclusions

This study investigated the drilling performance of MWCNT-reinforced CFRP nanocomposites through a data-driven, constrained multi-objective optimization framework. Rather than treating delamination as a competing objective, it was redefined as an engineering constraint derived from the first quartile of the experimental data distribution (*F_d_* ≤ 1.054), allowing the optimization to focus on simultaneously minimizing thrust force and torque while preserving structural integrity.

Regression-based surrogate models were developed for all three responses, with R^2^ values of 0.93 for thrust force, 0.64 for torque, and 0.77 for the delamination factor. For the torque model, insignificant terms were removed through stepwise regression, which improved model robustness. These models were integrated into NSGA-II and MOPSO algorithms to generate Pareto-optimal solution sets. Among the identified solutions, the high-productivity optimal point—corresponding to *w* = 0.93%, *v* = 70 m/min, and *f* = 0.10 mm/rev—yielded a thrust force of 49.36 N and a torque of 29.37 N·cm while satisfying the delamination constraint.

Both algorithms converged to consistent Pareto frontiers, with MOPSO achieving a slightly higher hypervolume (0.876 vs. 0.842) and NSGA-II providing a more uniform solution distribution (S = 0.114 vs. 0.138). MOPSO also demonstrated greater computational efficiency. The close agreement between the two algorithms strengthens confidence in the reliability of the identified optimal solutions.

Validation experiments confirmed that the surrogate models provide acceptable prediction accuracy across a range of near-optimal conditions, with thrust force errors remaining below 7.5% in most cases. The higher torque errors observed in some experiments are attributed to the anisotropic nature of the composite and the variability of chip formation during drilling, which are inherently difficult to capture through polynomial regression.

Overall, this study demonstrates that constraining delamination statistically rather than minimizing it directly yields a practical and reliable optimization framework for composite drilling. The proposed methodology is extendable to other composite systems and machining configurations involving conflicting performance objectives.

## Figures and Tables

**Table 1:** Control factors and level values used in the study.

Drilling Parameters	Symbol	Levels		
MWCNT Content	*w* (wt.%)	0	0.5	1.0
Cutting Speed	*v* (m/min)	25	50	75
Feed Rate	*f* (mm/rev)	0.10	0.15	0.20

**Table 2:** Statistical validation of the developed global surrogate models.

Response Variable	R^2^	Adj.R^2^	RMSE	*p*-Value
Thrust Force (*F*)	0.93	0.89	2.56	<0.0001
Torque (*T*)	0.64	0.59	4.24	<0.0001
Delamination (*F_d_*)	0.77	0.60	0.03	0.0016

**Table 3:** Regression coefficients and statistical validation of predictive models.

Model Terms	*F* (N)	*T* (N·cm)	*F_d_*
Intercept	26.416	9.68	1.257
*w*	−14.867	-	−0.189
*v*	−0.058	-	−0.001
*f*	447.5	252.062	−1.701
*w* ^2^	2.876	-	0.075
*v* ^2^	0.001	0.003	-
*f* ^2^	−791.778	-	5.733
*wv*	0.065	-	0.001
*wf*	9.6	-	−0.16
*vf*	−1.368	−2.918	0.006

**Table 4:** Control parameters determined for the NSGA-II and MOPSO algorithms.

Parameter	NSGA-II Setting	MOPSO Setting
Population/Swarm Size	100	100
Max Number of Iterations	500	500
Selection Method	Tournament Selection	Global Best
Crossover Rate/Inertia Weight	0.9	0.4–0.9 (Adaptive)
Mutation Rate/Repository Size	0.1	50 (External Repository)

**Table 5:** Experimental results of full factorial drilling tests: delamination factor (*F_d_*), thrust force (*F*), and torque (*T*) for all parameter combinations.

Exp. No	% *w*	*v* (m/min)	*f* (mm/rev)	*F_d_*	*F* (N)	*T* (N·cm)
1	0	25	0.10	1.156	56.8	24.64
2	0	25	0.15	1.128	69.59	45.38
3	0	25	0.20	1.138	76.08	46.3
4	0	50	0.10	1.102	54.93	33.33
5	0	50	0.15	1.123	67.09	36.45
6	0	50	0.20	1.154	70.98	36.16
7	0	75	0.10	1.071	54.98	29.94
8	0	75	0.15	1.056	63.02	34.82
9	0	75	0.20	1.164	64.6	32.95
10	0.5	25	0.10	1.038	57.7	28.81
11	0.5	25	0.15	1.068	64.62	37.65
12	0.5	25	0.20	1.064	72.54	46.36
13	0.5	50	0.10	1.06	49.97	29.5
14	0.5	50	0.15	1.072	59.66	31.05
15	0.5	50	0.20	1.088	67.15	51.65
16	0.5	75	0.10	1.097	54.19	33.39
17	0.5	75	0.15	1.071	51.87	33.94
18	0.5	75	0.20	1.067	61.12	32.95
19	1	25	0.10	1.025	49.53	29.23
20	1	25	0.15	1.033	60.64	39.1
21	1	25	0.20	1.064	66.37	48.37
22	1	50	0.10	1.078	45.91	29.55
23	1	50	0.15	1.047	60.58	27.73
24	1	50	0.20	1.039	63.01	28.42
25	1	75	0.10	1.038	47.32	26.88
26	1	75	0.15	1.053	57.94	30.26
27	1	75	0.20	1.117	61.21	37.66

**Table 6:** Effect of MWCNT content on drilling performance based on average experimental results.

MWCNT Content (wt.%)	*F_d_*	*F* (N)	*T* (N·cm)
0	1.12	64.23	35.52
0.5	1.07	59.87	36.14
1	1.05	56.95	33.02

**Table 7:** Pareto optimal solution sets and predicted response values for different drilling scenarios obtained via MOPSO algorithm.

Scenario	MWCNT (wt.%)	*v* (m/min)	*f* (mm/rev)	*F* (N)	*T* (N·cm)	*F_d_*
Minimum Thrust Force Oriented	1.0	64	0.10	48.03	28.48	1.052
Minimum Torque Oriented	0.84	50	0.10	49.08	27.79	1.047
High Productivity (Optimal)	0.93	70	0.10	49.36	29.37	1.054

**Table 8:** Statistical performance comparison of the NSGA-II and MOPSO algorithms.

Performance Metric	NSGA-II	MOPSO	Ideal Case
Hypervolume (HV)	0.842	0.876	Higher
Spacing (S)	0.114	0.138	Lower
CPU Time (s)	42.85	32.14	Minimum

**Table 9:** Validation and Error Analysis for All Control Experiments.

Exp.	Parameters (*w*, *v*, *f*)	*F* (Exp)	*F* (Pred)	Error (%)	*T* (Exp)	*T* (Pred)	Error (%)	*F_d_* (Exp)	*F_d_* (Pred)	Error (%)
1	0, 37.5, 0.13	66.66	63.77	4.33	37.48	32.44	13.45	1.183	1.125	4.90
2	0, 67.8, 0.13	55.65	59.82	7.49	28.78	30.52	6.04	1.2265	1.118	8.85
3	0, 30.14, 0.17	73.26	71.76	2.04	50.69	40.31	20.48	1.1512	1.134	1.49
4	0.5, 37.5, 0.13	57.91	58.90	1.71	32.46	32.44	0.06	1.139	1.057	7.20
5	0.5, 67.8, 0.13	52.28	55.93	6.98	30.91	30.52	1.26	1.16	1.066	8.10
6	0.5, 30.14, 0.17	66.10	66.84	1.12	40.68	40.31	0.91	1.207	1.06	12.18
7	1.0, 37.5, 0.13	55.92	55.47	0.80	36.92	32.44	12.13	1.087	1.027	5.52

## Data Availability

The original contributions presented in the study are included in the article, further inquiries can be directed to the corresponding author.

## Abbreviations

The following abbreviations are used in this manuscript:

CFRP	Carbon Fiber Reinforced Polymer
MWCNT	Multi-walled Carbon Nanotube
NSGA-II	Non-dominated Sorting Genetic Algorithm II
MOPSO	Multi-Objective Particle Swarm Optimization
*F_d_*	Delamination Factor
F	Thrust Force
T	Torque
w	MWCNT Weight Ratio
v	Cutting Speed
f	Feed Rate
R^2^	Determination Coefficient
$R_{adj}^{2}$	Adjusted Determination Coefficient
HV	Hypervolume
S	Spacing
RMSE	Root Mean Square Error
RSM	Response Surface Methodology
